# A 10-year prospectus for mathematical epidemiology

**DOI:** 10.3389/fpsyg.2023.986289

**Published:** 2023-06-09

**Authors:** Mark Orr, Henning S. Mortveit, Christian Lebiere, Pete Pirolli

**Affiliations:** ^1^Network Systems Science and Advanced Computing Division, Biocomplexity Institute, University of Virginia, Charlottesville, VA, United States; ^2^Department of Psychology, Carnegie Mellon University, Pittsburgh, PA, United States; ^3^Institute for Human and Machine Cognition, Pensacola, FL, United States

**Keywords:** cognitive modeling of human behavior, mathematical modeling and simulation, graph dynamical systems, epidemiology, psychology

## Abstract

There is little significant work at the intersection of mathematical and computational epidemiology and detailed psychological processes, representations, and mechanisms. This is true despite general agreement in the scientific community and the general public that human behavior in its seemingly infinite variation and heterogeneity, susceptibility to bias, context, and habit is an integral if not fundamental component of what drives the dynamics of infectious disease. The COVID-19 pandemic serves as a close and poignant reminder. We offer a 10-year prospectus of kinds that centers around an unprecedented scientific approach: the integration of detailed psychological models into rigorous mathematical and computational epidemiological frameworks in a way that pushes the boundaries of both psychological science and population models of behavior.

## 1. Introduction

Prospectus (noun) means an offering that provides a forward (pro) view (spectus), one that is typically used to show potential investors what financial gains might be made of shares in a product or company and a look at the features of said company that should instill confidence in the investor. We offer a prospectus of kinds: a view of potential societal gains if we invest in developing the next generation of at-scale, agent-based epidemiological simulation modeling. This kind of modeling will build scientific understanding of at-scale, meaningful, impactful, and real-world dynamics of human behavior across technological, social and physical networks or contexts. Although our prospectus focuses on the dynamics of infectious disease (e.g., SARS-CoV-2, Ebola, Influenza, Monkeypox), it applies equally to social phenomena in general and beyond scientific interest alone—understanding, and ultimately intervening on large scale human behavior during times of crises and major sociotechnical system-level shocks is a key problem central for public health, national security, climate change, economic stability, and disaster preparedness.

A major component of this prospectus relies on developing novel ways in which human behavior is represented in at-scale, agent-based simulations. We have seen some interesting work over the past decade that addressed the effects of human behavior on infectious disease dynamics (Funk et al., 2015; Verelst et al., 2016), but nothing that entails the integration of detailed psychological constructs, assumptions and models. Because of the complex and dynamic nature of epidemiological contexts, well-detailed and theoretic (as opposed to just descriptive and statistical) explanations should derive from theories in psychology, economics, neuroscience, and the cognitive sciences. To complicate matters, our prospectus puts tight constraints on how we use psychological theory and constructs: (i) the psychological theory must be in formal terms precise enough for implementation in computer code and definable as a mathematical object, and (ii) the implementation of such theory must factor in the degree of computational complexity in time and space.

**Figure 1:**
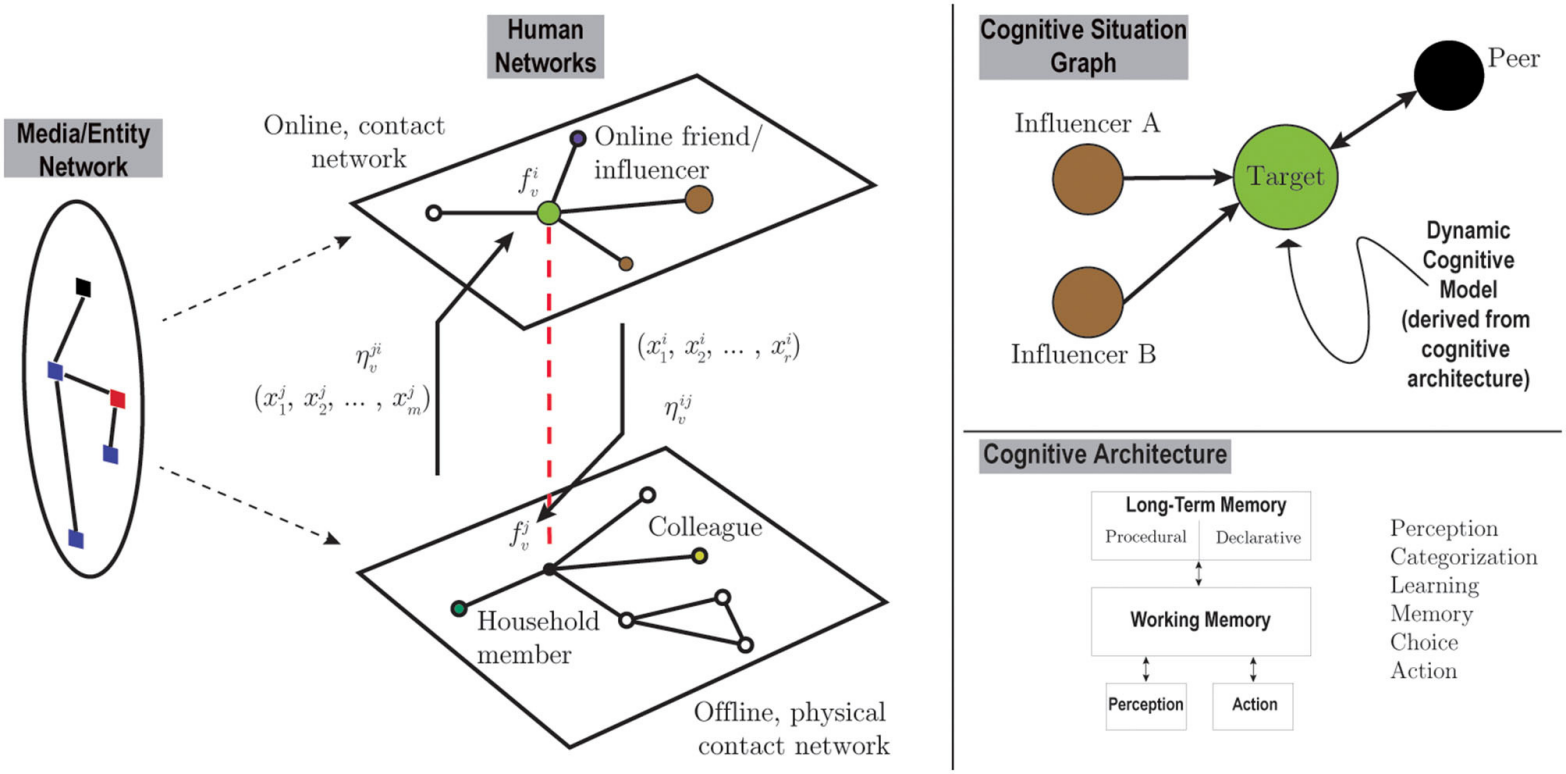
The 10-year prospectus imagined graphically shows the larger system graph illustrated on the left captured through coupled, co-evolving networks, possibly including mass media. Transfer functions (e.g., η_1_ and η_2_) govern how states associated to a different network layer (e.g., online) may influence dynamics in another network layer (e.g., physical contacts). A cognitive situation graph is illustrated in the top right, capturing dynamics at a compact level for the various agent classes present in the system network. Essential to this 10-year prospectus is the invocation of human cognitive architectures to realistically constrain mathematical models of system-level behavior.

Our prospectus not only provides methods for developing richer representation of psychological processes and representations in agent-based simulations but, critically, includes a formal rigorous mathematical framework, Graph Dynamical Systems (**GDS**), for both designing and understanding the behavior of at-scale, complex systems. Graph Dynamical Systems is a framework for efficient and accurate design of at-scale agent-based models, one that is formal and rigorous, flexible, and maps well to modern computing hardware (for scaling purposes). Figure 1 puts these two notions together (figure details are provided in the next section).

## 2. Scientific framework

The foundation of our 10-year prospectus is a scientific framework for analysis, modeling and simulation of systems that span multiple scales and networks. It captures the co-evolving, coupled networked systems via what we call a *system graph* composed of multiple sub-systems. Each sub-system (or component) is a separate GDS and is described by a network *G* over a set of vertices *V*. For each vertex *v* of this sub-system, there is a state *x*_*v*_ and a function *f*_*v*_. We denote the system state as a sequence *x* = (*x*_*v*_1__, *x*_*v*_2__, …, *x*_*v*_*n*__), or simply *x* = (_*x*_*v*_)*v*_, and we write *f* = (_*f*_*v*_)*v*_ for the corresponding vertex-indexed sequence of functions. The function *f*_*v*_ captures the characteristics of that vertex and governs how the state *x*_*v*_ of vertex *v* evolves with time taking as input the vertex's own state as well as the state of its neighbors in the network *G*. An update mechanism or schedule governs how all the vertex functions *f*_*v*_ with *v*∈*V* assemble to generate the dynamics of the sub-system. In summary, each component system is described by a network *G*, a sequence of vertex states *x* = (_*x*_*v*_)*v*_, vertex functions *f* = (_*f*_*v*_)*v*_, and an update mechanism. The complete system is modeled using a collection of graphs as above. We thus have graphs $G={\left(G_{i}\right)}_{i}$ with matching vertex functions $F={\left(f^{i}\right)}_{i}$ where $f^{i}={\left(f_{v}^{i}\right)}_{v}$, and a corresponding (total) state $X={\left(x^{i}\right)}_{i}$ and $x^{i}={\left(x_{v}^{i}\right)}_{v}$ is the state in network *G*_*i*_. In addition, there will be a collection of transfer functions η between each pair of networks, see Figure 1, that captures how a vertex state *x*_*v*_ on one graph may influence to the same vertex state on another graph.

The mapping to psychological constructs is straightforward. The functions in the sequence ${\left(f_{v}^{i}\right)}_{v}$ capture the psychological models relevant for all individuals represented and interacting on the graph *G*_*i*_. For the individuals present in multiple graphs, the transfer functions η relate the psychological models on different graphs (e.g., between *G*_*i*_ and *G*_*j*_), something that can capture unique transfer functions for specific individuals and are denoted by ${\left(\eta_{v}^{ij}\right)}_{v}$. The transfer functions can be directional, to mean that the mapping from ${\left(x_{v}^{i}\right)}_{v}$ to ${\left(x_{v}^{j}\right)}_{v}$ is not necessarily equal to that of ${\left(x_{v}^{j}\right)}_{v}$ to ${\left(x_{v}^{i}\right)}_{v}$; in notation this is ${\left(\eta_{v}^{ji}\right)}_{v}$ and ${\left(\eta_{v}^{ij}\right)}_{v}$ for the former and latter, respectively.

To put this in real-world terms, consider the example shown on the left panel of Figure 1; each of the two component graphs (e.g., an online contact network such as Twitter and a physical, real-world contact network) will encode vertices as individual people and edges as possibilities of interactions among them. Some individuals are represented on both GDS in which case we map the relation between GDS (or contexts) with the transfer functions. An example of a non-symmetric transfer function, for example, would be that beliefs learned on Twitter (*G*_*i*_) might transfer to the mechanism of action (wear a mask) in the physical world (*G*_*j*_) via $\eta_{v}^{ji}$, a relation that may not be reciprocated by $\eta_{v}^{ij}$.

The identification of individuals into types is an important feature of our system graph. Thus, for a given context and graph, individuals are identified using a suitable equivalence relation (all individual vertices fall under an equivalence class, e.g., all vertices who are targets of a misinformation campaign are of one equivalence class). The vertices of what we call the cognitive situation graph (upper right of Figure 1) are the equivalence classes under this relation, with edges being induced from the full network of the given context. Referring to the upper right of Figure 1, “Target” may represent all those who are vaccine neutral, while “Influencer A” may represent the collection of people who are against vaccine, both in an online network. Note that such a class may be present in multiple situation graphs and networks contexts (e.g., persons against vaccine in online and offline networks). Deriving models for the agents of a situation graph (i.e., the equivalence classes of agents) is both an efficient and practical approach for embedding cognitive models into graph representations. A similar approach was used in our work (Barrett et al., 2013) in a different domain (disaster preparedness), but the systems here are more complex, and also have more complex coupling captured by the transfer functions.

In summary, our scientific framework speaks to a particular kind of scientific application, what we call *constrained multiscale explanation*, an approach that affords explanation of dynamic phenomena in multiplex social systems at multiple levels of scale and in a way that is constrained–in design and for analytic purposes—by a formal mathematical treatment. Our prospectus was developed with the recognition that building scientific explanations for phenomena in at-scale social systems must consider different classes of scientific issues: within disciplinary, cross-disciplinary and the unique issues implied by the larger social system. Further, our prospectus realizes mechanisms at different levels of scale: individual psychological processes, the interaction between individuals and social groups characterized by sociology and economics, and large-scale contextual and emergent system-wide processes.

The remainder of this article will explore (i) psychological theory instantiated as cognitive models followed by (ii) graph dynamical systems, (iii) the integration of cognitive modeling with graph dynamical systems, and (iv) the simulation of infectious disease dynamics in a way that incorporates cognitive models of individuals and is informed by graph dynamical systems.

## 3. Psychological theory, cognitive architectures, and cognitive modeling

Psychological theory provides (i) theoretical psychological mechanisms, (ii) insights into the external social & environmental (contextual) cues/stimuli/communications that drive behavior, and (iii) methods of measurement for theoretical constructs. Besides rational/cognitive models—e.g., Theory of Planned Behavior/Reasoned Action (Ajzen, 1991)—from social psychology, theory can integrate insights from behavioral economics (Boyd, 2020), habit theory (Zhang et al., 2022), as well as the role of emotions for identification of the features and cues in the social context that shape behaviors (van Doorn et al., 2015). It is precisely these kinds of theory that should be integrated into at-scale, agent-based simulations of infectious disease.

In our scientific framework, psychological theory is wedded to the vertex state functions (_*f*_*v*_)*v*_ and the transfer functions (_η_*v*_)*v*_. Thus, its representation must yield something both mathematically tractable and computationally implementable while retaining important theoretical commitments relevant to behavior in epidemic contexts. However, the psychological literature, primarily in relation to social psychology, is limited in terms of formal mathematical or computational theory for our purposes. For example, computational models of attitude formation and change have been developed as stylized, and highly abstracted analogs to (i) hypothetical, (ii) specific tightly-controlled experimental, or (iii) survey contexts (Van Overwalle and Siebler, 2005; Overwalle, 2007; Monroe and Read, 2008; Dalege et al., 2016, 2018; Galesic et al., 2021). It is unknown to what degree these models are relevant for real-world behaviors and decisions that are social in nature and relevant for infectious disease dynamics. There exist sporadic calls in the public health literature for the integration of behavior change theory with computational psychology in public health (Orr et al., 2013, 2017, 2019; Orr and Plaut, 2014; Pirolli, 2016; Orr and Chen, 2017) but these suffer from similar issues to those found in the social psychological literature. A more domain-general approach is needed.

Our scientific framework, in contrast, invokes the notion of cognitive architectures as the basis for models of behavior implied in infectious disease. As is typical, we reserve the term cognitive modeling to refer to the class of models of behavior that are constrained by cognitive architectures and attempt to model how the human mind drives behavior (as opposed to modeling human-like behavior in any way possible). But what are cognitive architectures and what is the value of cognitive modeling in terms of infectious disease epidemiology?

Cognitive architectures, as computational implementations of unified theories of cognition, provide predictive quantitative constraints on human behavior across all fields of human activity. A prominent example is ACT-R, a highly modular cognitive architecture that was designed specifically for the purpose of cognitive modeling. It is composed of a number of modules (e.g., procedural and declarative memory, perception and action) that operate asynchronously through capacity-limited buffer interfaces (Anderson, 2007). Each module is in turn composed of a number of independent mechanisms, typically consisting of symbolic information processing structures combined with equations that represent specific phenomena and regularities (e.g., power law of practice and forgetting). The architecture includes a number of learning mechanisms to adapt its processing to the structure of the environment. The combination of powerful mechanisms together with human capacity limitations (e.g., working memory, attention, etc.) provides a principled account of both human information processing capabilities as well as cognitive biases and limitations.

An important feature of ACT-R, in comparison to other cognitive architectures, is that it has sustained decades of validation against human experimental data. This feature, we argue, is imperative for epidemiology because it grounds at-scale epidemiological population agent models in the psychological and cognitive sciences. Our prospectus advocates the use of such grounded cognitive architectures as the basis for developing agent-level cognitive models in epidemiological simulations.

Many efforts have been made to build cognitive models of human behavior across a wide range of applications, ranging from simple psychology experiments to decision making to complex dynamic task environments (e.g., decision making, sense-making, game playing, and interactions over social networks); see for example (Anderson, 2022). The practice and use of building cognitive models varies in terms of the scope of architectural components or modules. Some cognitive modeling efforts have leveraged modeling frameworks to specify knowledge-level structures and processes as additional constraints. For instance, instance-based learning (IBL) assumes that decisions are based on experience, leveraged through memory mechanisms (Gonzalez et al., 2003) which can be implemented using components of the ACT-R cognitive architecture. The notion of accountable modeling attempts to cleanly separate aspects of human performance that are based on theoretical cognitive constraints from model parameterizations that reflect other factors such as procedures and task environment. The former are assumed to be invariant across tasks and contexts (Reitter, 2010).

A key feature of cognitive models in respect to epidemiology is their generative and predictive nature. Thus, they can be used to optimize behavior-change interventions, both in design or as an online surveillance aid. For example, our recent work has integrated cognitive modeling using ACT-R with network simulations of population responses to public health messages of non-pharmaceutical interventions and their impact on epidemiological spread (Pirolli et al., 2020, 2021); another similar example is the modeling of the effects of (in)coherence of messaging and sources on credibility (Liao et al., 2012).

Our prospectus, in short, leverages cognitive models constrained by a grounded cognitive architecture to provide a single unified computational formulation of disparate psychological and other behavioral theories, e.g., the integration of multiple factors into a single predictive theory.

## 4. Graph dynamical systems

In this section, we provide a brief formal description of the Graph Dynamical Systems approach (readers may find it useful to refer to Section 2). We then introduce the reasoning behind using this framework in the context of at-scale, agent-based simulations of infectious disease, something that applies to many analogous systems. To preface of our reasoning, we note that at-scale, agent-based simulation approaches to human-relevant infectious disease dynamics are quite complex. GDS provides several features for both the analysis of such dynamics and the design of simulation systems without which, we claim, would render it unreasonable to make any substantive scientific or practical claims. The key focus of our prospectus, to integrate psychological processes into such models, brings such need into sharp relief.

### 4.1. Formal description

The mathematical and computational theory of GDS (see, e.g., Goles and Martinez, 1990; Barrett and Reidys, 1999; Barrett et al., 2000b, 2001, 2003a,d, 2006, 2009; Mortveit and Reidys, 2001, 2007; Rosenkrantz et al., 2015) is largely concerned with the formal abstraction of dynamics evolving over networks. For this, the theory is generally focused on finite state sets (e.g., {0, 1}) and specific update mechanisms used to assemble local dynamics of agents into global dynamics of the complete system. Formally, a sequence of vertex function (_*f*_*v*_)*v*_ indexed by the agents will, by applying an update scheme *U*, assemble to a map $F_{U}:K^{n}\to K^{n}$ where *K* is the state set of each agent. For example, for a parallel update scheme with *n* agents/vertices, we have


$$\begin{array}{l} F_{U}\left(x=\left(x_{1},\ldots ,x_{n}\right)\right)=\left(f_{1}\left(x\right),\ldots ,f_{n}\left(x\right)\right). \end{array}$$

Here the variable dependencies in the functions *f*_*v*_ reflect the network *G*. Existing mathematical and computational theory is concerned with how structural properties of the functions *f*_*v*_, properties of the network *G*, and the choice of update mechanism translate into properties of the system dynamics of the system map *F*_*U*_. All standard questions and topics such as stability and control are studied.

### 4.2. Rationale for GDS

The GDS framework has been central for analytics and design of simulation models for co-evolving networked systems in prior work for more than two decades, having been applied to epidemiological studies, evacuation scenarios, and large-scale models for resilience in socio-technical system at large (Barrett et al., 2000a, 2013; Cedeno-Mieles et al., 2018; Adiga et al., 2019; Chen et al., 2020a,b; Islam et al., 2020; Meyur et al., 2020; Swarup and Mortveit, 2020; Wang et al., 2020). The framework of GDS, and in particular the notion of vertex function, was designed specifically to support (i) precise modeling of networked systems, while (ii) being amenable to mathematical and computational analysis, and at the same time (iii) mapping well to high performance computing hardware (Barrett et al., 1998, 2000b, 2001, 2003d; Barrett and Reidys, 1999; Atkins et al., 2008; Laubenbacher et al., 2009). Each of these features has important implications for the feasibility of use of at-scale, agent-based epidemiological simulations.

The key distinguishing factors at the level of behavioral aspects in our prospectus include (i) the vertex functions, derived from cognitive models, are significantly more complex, (ii) the dependencies on other agents within a network (i.e., the network structure) are not that well known, and (iii) agents may only be privy to partial observations of state of the agents with whom they interact, and the latter party may additionally choose to apply deceptive strategies when revealing their partial state. To address (i)–(iii), significant extensions of theory and structure of GDS are needed both at the level of fundamental theory and for integration within the design of simulation models, as detailed in the next section. In plain terms, vertices that represent human behavior as cognitive models pose challenges to the GDS framework. We provide a sketch of how this might be accomplished in the next section.

## 5. Integration of cognitive architectures and graph dynamical systems

**Figure 2:**
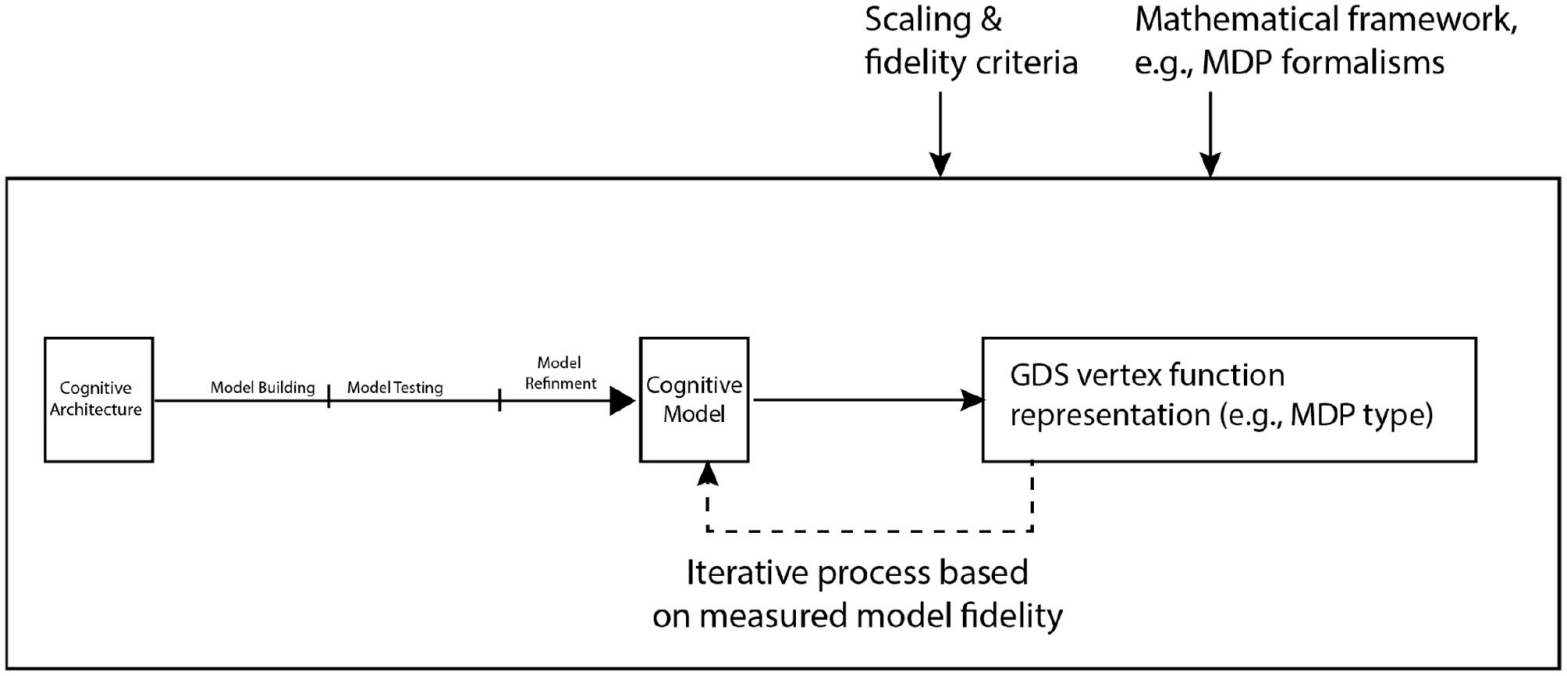
A major component of our 10-year prospectus is to develop and design vertex functions for the GDS framework from cognitive first principles (i.e., derived from or constrained by a human cognitive architecture). The left portion above shows the development from cognitive architecture to cognitive model. The dotted-arrow represents an iterative process that is designed to vary the degree of abstraction (more abstraction means less fidelity) in the mathematical representation of an agent's cognitive model. Scaling criteria are considered in respect to the time and space complexity of computations on the graph; for large graphs with high-fidelity vertex functions, this may be a serious consideration. The mathematical frameworks for representing vertices are various and may be explored as part of the development of a GDS formalism.

Our prospectus follows the design illustrated in Figure 2 for modeling vertex functions. Key features of our approach are: (i) it is applicable across agent classes, (ii) the choice of framework for representing vertex functions (e.g., Markov decision process, Boolean functions) is flexible, and (iii) it provides a mechanism for quantifying how well vertex functions approximate the domain (e.g., cognitive) model, and thus a means for assessing the balance between fidelity of agent representations with computational scaling in the simulation models (Figure 2 caption provides further details on these features). More broadly, our approach provides *a generic approach for integrating domain knowledge of a context or situation graph into a computational framework for networked systems while keeping track of the fidelity of the mapping from domain knowledge and expertise (e.g., the kind of cognitive model to represent) to vertex functions*.

In the diagrams of Equation (1) the map β_*v, c*_ is the cognitive model for agent *v* in the context *c*, while ${\left(\eta_{v,\left(c,c^{\prime }\right)}^{\prime }\right)}_{c^{\prime }}$ is the collection of transfer functions factoring into the cognitive model for agent *v* in the context *c* impacted by other contexts *c*′. These are formalizations of the cognitive models of behavior. While one would typically expect that the cognitive models for agents β_*v, c*_ to be computationally heavy, one may in principle use a suitable update mechanism *U*′ to assemble these functions across networks to form the full system model *M*_β, *U*_ driving the system dynamics as illustrated at the top in the rightmost diagram of Equation (1).


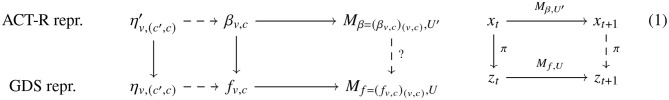


The cognitive behavioral components can be mapped into a GDS form by constructing a general approach to (i) translate cognitive models into GDS vertex functions β_*v, c*_→*f*_*v, c*_, (ii) match this for the associated transfer functions $\eta_{v,\left(c^{\prime },c\right)}^{\prime }\to \eta_{v,\left(c^{\prime },c\right)}$, and (iii) develop metrics for how well *f*_*v, c*_ captures β_*v, c*_ in preparation for assessing how well the system map at the GDS level (i.e., *M*_*f, U*_ in Equation (1)) captures the composed cognitive model $M_{\beta ,U^{\prime }}$. It is worth pointing out that the state space for the cognitive model and for the GDS translation need not be the same, thus there may be a mapping π connecting the two. Similarly, while it is likely that the update mechanism *U* and *U*′ for the assembly of local-to-global dynamics may be the same in both cases, this is not generally required (Róka, 1999).

To develop the mapping β_*v, c*_→*f*_*v, c*_ from cognitive models into suitable vertex functions, future work could leverage prior work (Sycara et al., 2015) as a starting point. In this prior work, the cognitive model abstracted human performance and was in turn abstracted into an analytical framework captured as a Markov decision process (MDP). The mechanisms of the ACT-R cognitive architecture provided additional theoretical constraints on a limited amount of human data, especially when developing personalized models (see Cranford et al., 2020). In turn, the representation of the IBL process in ACT-R declarative memory provided additional constraints on the states of the MDP. Thus, the framework for Markov decision processes (MDP) can be used to represent vertex functions capturing cognitive models that are represented and calibrated using ACT-R (Sycara et al., 2015).

MDP is a natural framework for modeling stochastic processes or phenomena with inherent uncertainties, and also lend themselves well to controlling computational scaling by (i) adapting the resolution of selected states (e.g., more fine-grained states) and (ii) by an increase in fidelity resulting from modifying the dimension of the agent's state space used in its vertex function representation (see, e.g., Barrett et al., 2006; Rosenkrantz et al., 2015) for a joint characterization of the boundary for which many GDS problems become intractable formulated in terms of the network and vertex function complexity.) In addition to MDPs, future work could consider deterministic Boolean- and finite state space representation for vertex functions (Mortveit and Reidys, 2001, 2007). This could be used for (a) simplified models, (b) studying scenarios for large systems involving scaling, (c) and for model validation and verification of the simulation framework. This allows for bridging from existing mathematical and computational theory of GDS (Goles and Olivos, 1980, 1981; Goles-Chacc et al., 1985; Goles and Martinez, 1990; Barrett and Reidys, 1999; Barrett et al., 2000b, 2003a,b,c,d, 2006; Mortveit and Reidys, 2001, 2007; Laubenbacher and Pareigis, 2003, 2006; Macauley and Mortveit, 2008, 2009, 2011; Laubenbacher et al., 2009; Rosenkrantz et al., 2015). We remark that it is also possible to consider algorithmic or procedural representations for vertex functions.

## 6. Integration into at-scale systems

The methodological considerations when building an at-scale agent-based simulation are vast for any domain of study. Several well-vetted, industry-grade platforms exist for such efforts (e.g., AnyLogic, REPAST) as do academic, in-house enterprises [e.g., Matrix (Bhattacharya et al., 2019); EpiHiper (Machi et al., 2021)].

Our prospectus does not advocate methods or platforms. Instead, we offer a framework for incorporating psychological theory into at-scale agent-based simulations. Naturally, our framework increases the complexity of the system components and, potentially, the dynamics of the system. GDS provides a mathematical framework for taming such complexities.

## 7. Summary

The overarching offering of our 10-year prospectus is a more nuanced understanding of the implications of human behavior on the dynamics of infectious disease. The primary scientific advance will stem from the coupling of high-fidelity models of human behavior, derived from the domain-general cognitive architecture, and the rigorous mathematical framework of GDS for understanding complex system dynamics. This, we surmise, will form the foundation for more detailed, realistic and usable agent-based simulations of infectious disease in human populations.

Is this 10-year prospectus feasible? It leverages long-standing results and methods from mathematical and computational epidemiology, human cognitive architectures, and graph dynamical systems into a convergent approach. We think the technological and scientific advances have set the stage to tackle some of the more difficult issues that implicate human behavior, e.g., fatigue effects, trust/credibility, attitudinal polarization, social learning; utility-satisficing, etc. and how these are interdependent with proximal behaviors that drive disease dynamics, e.g., non-pharmaceutical intervention/protection, vaccination, and medical intervention.

## Data availability statement

The original contributions presented in the study are included in the article/supplementary material, further inquiries can be directed to the corresponding author.

## Author contributions

MO, HM, CL, and PP contributed to conceptualization and design of the content and writing of the content. All authors contributed to the article and approved the submitted version.
